# Beyond Collaborative Learning: a Comparison of Small Groups in Face-to-Face and Online Settings

**DOI:** 10.1007/s40670-024-01983-4

**Published:** 2024-01-15

**Authors:** Xiaomei Song, Michael Elftman

**Affiliations:** 1 School of Medicine, Case Western Reserve University, 9501 Euclid Avenue, Cleveland, OH 44106-7503 USA; 2 College of Medicine, Central Michigan University, Mount Pleasant, USA

**Keywords:** Collaboration, Learning community, Virtual, Online, Face to face, Small group, CBL, TBL, Peer evaluation

## Abstract

Small group-based instructional approaches such as case-based learning (CBL) and team-based learning (TBL) are widely used in medical education to promote collaboration and team learning. During the pandemic, many medical schools shifted from face-to-face instruction to online settings. While CBL/TBL are intended to foster collaborative skills, it is unclear how its use evolves in an online setting and whether the online setting impacts students’ perceptions and behaviors in collaboration. This study examined how the change from in-person to online CBL/TBL impacted students’ collaboration. We used a mixed-methods sequential design, first collecting and analyzing retrospective cohort quantitative data with the Class of 2023 through peer evaluation surveys followed by six focus groups. Students’ assessment of their peers was generally positive. Nonparametric testing showed significant differences for two questions with less positive perceptions in the virtual setting compared to when students had in-person CBL/TBL. The focus group results identified several themes related to collaboration and learning communities. In the virtual setting, students not only lost collaboration opportunities with their group members in CBL/TBL, but also learning opportunities and social connections with other groups and the community as a whole. Virtual learning environments may have presented challenges for collaborative learning and the establishment of a sense of community.

## Introduction

Collaboration is an important skill for medical professionals [1]. Developing proficiency in collaboration entails affective aspects of learning, and students achieve learning outcomes by responding to and engaging with their peers through teamwork. Small group-based instructional approaches such as case-based learning (CBL) and team-based learning (TBL) are widely used in medical education to promote collaboration and team learning in the application of higher order thinking to complex problems [2, 3]. These small groups create intentionally structured learning communities that foster and maximize the individual and shared learning of group members. During the COVID-19 pandemic, many medical schools shifted from face-to-face instruction to using online settings. This shift has highlighted the need to critically assess the effectiveness and limitations inherent in the virtual setting [4]. While CBL and TBL are intended to foster learning and support the development of collaborative skills, it is unclear how its use in an online setting evolved and whether the online setting impacted students’ perceptions and behaviors in collaboration. As such, this study examined how the change from in-person to online CBL and TBL impacted students’ collaboration.

## Collaboration

Collaboration has drawn much attention since Dewey’s explorations into the social nature of learning. Collaborative learning theory has been further developed and can be rooted in Lev Vygotsky’s constructivist perspective the “zone of proximal development” [5, 6]. He explained how a person can learn from interactions with others that were more advanced within the zone of proximal development. It is a powerful approach in which individuals work together to build knowledge and achieve a common goal. Research examining the collaboration process concludes that adopting a collaborative approach enables individuals to develop higher order thinking skills and professional behaviors that have a greater impact on learning outcomes [7, 8]. For example, Frykedal and Chiriac investigated students’ inclusive and collaborative processes in group work [9]. They concluded that students’ negotiation of their individual responsibilities, active participation in the discussions around working structure and the task, and the investigative and analytical parts of discussions promote their inclusive and collaborative processes.

Instructional methods such as CBL and TBL are intended to facilitate students’ learning by encouraging the practice of researching, comprehending, and evaluating information about medical concepts, and defending and making logic arguments about scientific decisions [3, 10, 11]. Those methods also promote the distribution of knowledge where students work collaboratively to ask/answer questions, share their results, and perform on questions or test items to create a collaborative community of learners. CBL and TBL challenge students to develop supportive relationships and promote a strong sense of shared responsibility regarding group performance: we all succeed, or we all fail.

CBL and TBL have been adopted into the integrated curriculum of Central Michigan University’s College of Medicine (CMED) since 2013. After students matriculate at CMED, they are randomly assigned to small groups of 7–9 students considering gender to balance the groups. All students are reassigned to new groups after the first year. During the first 2 years of its curriculum, CBL and TBL are operated in all organ system courses using a standardized approach. Individual students prepare for TBL activities by reading pre-assigned material before participating in individual- and group-based activities each week. The same groups also participate in CBL by collectively working through patient cases before participating in additional TBL sessions based on each case. These combined CBL/TBL activities occur three times each week in all organ system courses.

Like many schools, the COVID-19 pandemic brought the unprecedented closure of nearly all face-to-face instructional delivery modes at CMED, including CBL/TBL. Several studies have found that online learning is no less effecting than face-to-face instruction for the acquisition of knowledge and skills [12, 13]. However, the online setting is known to present unique challenges for the development and functioning of student teams [14]. While it is well-documented that CBL and TBL foster the development of collaborative skills, there is limited research investigating the extent to which these benefits can be replicated in virtual settings [4]. The effectiveness of online small group settings in achieving comparable outcomes remains unclear, particularly when considering the challenges posed by limited facial cues and physical visualization. This study addressed two research questions: (1) are there significant differences in students’ perceptions and behaviors between face-to-face and online collaboration? (2) if such differences exist, how does the shift from face-to-face to virtual learning affect students’ collaboration?

## Methods

This study was determined to be exempt from institutional review board (IRB) review by CMU’s IRB. We used a mixed-methods design, first collecting and analyzing quantitative data through peer evaluation surveys followed by focus group interviews [15, 16]. In this study, the quantitative stage primarily focused on occasions where small groups interacted with each other to complete tasks during the CBL/TBL sessions. Whereas the quantitative stage was limited to students’ interactions during the instructional period, the qualitative stage probed students’ interactions more broadly and explored a larger learning context beyond the course. The quantitative data collection and analyses were completed prior to the qualitative stage. The results of the quantitative data were integrated and used to inform the design of the semi-structured interview protocol with a broader scope used in the qualitative stage. As such, the qualitative stage was emphasized to explain the initial quantitative results and expand our understanding of the phenomenon in more depth.

This study included medical students in the CMU College of Medicine’s class of 2023. We began by comparing retrospective peer evaluation data from two 6-week Year 1 organ system courses: the Reproductive course that was delivered entirely through face-to-face instruction in early 2020 and the Renal/Endocrine Wellness & Disease course conducted entirely online from April to June 2020. These two courses are part of our preclinical organ system-based curriculum and use similar instructional methods including a combination of CBL, TBL, and large group (lecture-style) sessions. In the first course, these sessions were held face-to-face. In the second course, students participated in the same types of sessions, but they were held virtually using Webex. After each course, all 101 students completed our standard peer evaluations, which included five items that asked students to assess their peers’ skills at collaboration using a 5-point Likert scale. Responses were analyzed using SPSS. The average scores for each item were calculated. Because of the violation of normality of the responses, the nonparametric Wilcoxon signed-rank test was chosen to compare peer evaluation scores in the two settings.

To further explain our quantitative findings, we conducted focus groups to explore students’ perceptions in depth. Based on the quantitative findings and previous literature [17], a semi-structured interview protocol was designed asking students to provide their interpretations of survey results as well as their personal experiences and reflections of collaborative learning in small groups in the two settings. All students from the class of 2023 were invited to participate through emails. We used a purposeful sampling approach and considered gender. Six groups with 27 students were recruited with 11 males and 16 females. All focus groups were conducted by one research team member who does not directly interact with students in the educational program. Focus group interviews were recorded and transcribed verbatim. The transcription was analyzed using a thematic inductive coding method [18]. Two researchers developed the initial draft coding scheme, followed by an iterative process of modifying and recoding before producing a finalized coding scheme.

## Results

### Quantitative Findings

Cronbach’s alpha internal consistency reliability coefficient was calculated for the five 5-point Likert scale items in both courses, resulting in satisfactory reliability (*α* = 0.939 and 0.951, respectively). These findings indicate that the items effectively captured the dimension of collaboration within the courses. Students’ assessment of their peers was generally positive and the average scores for face-to-face were comparable to or higher than scores in the virtual setting. As showed in Table 1, nonparametric testing indicated significant differences for two collaboration questions between two settings: “demonstrates a good balance of active listening and participation” (*Z* =  − 7.203, *p* < 0.001) and “asks useful or helpful questions” (*Z* =  − 7.386, *p* < 0.001). Students’ responses for these two items were significantly lower in the virtual setting compared to when students had in-person CBL/TBL.

**Table 1:** Quantitative results

**Survey item**	**Setting**	**Mean**	**SD**	***Z***	**Asymp. Sig. (2-tailed)**
Demonstrates a good balance of active listening and participation	F2F	4.64	0.326	− 7.203	< 0.001
	Virtual	4.19	0.484		
Asks useful or helpful questions	F2F	4.71	0.289	− 7.386	< 0.001
	Virtual	4.25	0.434		
Shares information and personal understanding	F2F	4.78	0.242	− 1.347	0.178
	Virtual	4.76	0.230		
Identifies references with relevant information	F2F	4.77	0.263	− 1.089	0.276
	Virtual	4.75	0.250		
Helps team stay focused and on track	F2F	4.64	0.338	− 0.781	0.435
	Virtual	4.67	0.289		

After completing these quantitative analyses, focus groups were conducted to explain the quantitative results, in particular, these significant results, and further probe students’ perceptions and experiences in two settings. While interview questions gave special attention to the quantitative findings such as active listening and responding, participants were provided the opportunity to reflect on their personal experiences in depth beyond the class hours during CBL/TBL and discuss various areas related to collaboration in small groups.

### Qualitative Findings

Although the study initially aimed to investigate collaborative learning, two prominent themes emerged from the focus groups: collaboration and learning communities. In our codebook, collaboration was defined as the sharing of knowledge within the group during class time, while learning communities encompassed other aspects beyond knowledge sharing within small groups, formally and informally. These themes provided valuable insights into the broader dimensions of interaction and connections with the group and across the group.

In terms of collaboration, the interviewees confirmed the significant decline regarding the quantitative findings of “demonstrates a good balance of active listening and participation” and “asks useful or helpful questions” due to various reasons. The participants also perceived no major changes related to the other three items in the two settings, which were consistent with the quantitative results. The following codes emerged from the interview data: (1) lack of visual cues, (2) changed goals, (3) adaption to online learning, and (4) group dynamics. First, the students elaborated on how the online format interfered with communication and collaboration. They frequently mentioned that the lack of facial cues when their teammates did not use cameras created issues for proper and effective interactions. With cameras off, nonverbal behaviors such as body movements and posture, facial expressions, and eye contact were all lost, which made it difficult to ask questions and understand each other. The following student explained:

TBL is really hard, I feel like. And the case was really hard with the 2nd group [in Year 2], particularly because we just haven’t had that experience with each other initially, you know, you don’t want to offend anyone when you can’t see their face, or you don’t want anyone to take anything the wrong way. And then, you know, if two people try to talk at the same time, you can’t hear each other. So, it’s kind of like, “no, you go. I go.” So, there’s like an awkwardness to it. Um, so, yeah, online was kind of difficult. I definitely say. (S3)

Second, the CBL/TBL groups changed their goals and focused on the rapid completion of cases and questions instead of insightful discussions. Online CBL/TBL enabled students to become more efficient as they used two-screen setups at home, shared information easily, and discussed major questions without being repetitive; however, the participants realized that efficiency was often at the expense of group discussions and interactions, and they lost opportunities for in-depth discussions. The participants stated that “there was like less distractions” (S14) and asked fewer questions that “would spark the conversation” (S2). The situation became “more, just kind of like, filling it out and getting off” (S20) and “we’re kind of rushing through cases and not taking the full hour that we’re given” (S1). For instance,

We kind of like stopped collaborating as much. The dynamic was still similar because my group was just very involved in the beginning with TBL and case in general. And we kind of stayed that way online, but I think it kind of made people want to discuss big topics less. And we just kind of moved on from things quicker instead of actually working it out, and we didn’t talk to professors as much because we didn’t want to like, bring them into our [virtual] room or whatever it was. (S16)

Consequently, the in-depth discussions and meaningful conversations about cases and questions to maximize their learning diminished. As one student described, “You lose that…being able to explain something to someone else strengthens your own understanding of the concept as well as them being able to kind of talk through it with you strengthens their understanding” (S3).

Third, learning was vastly shifted from group responsibility to individual accountability in the online setting. The students reported they had to change their learning approaches, for example, “it’s been more on me to, to learn certain things that I would otherwise hear from somebody else, or I would have figured out a little bit easier” (S9). They modified their strategies as “you have to step up a little bit [by self-studying]. Because you don’t have many people kind of holding you accountable” (S21). The following student elaborated on her learning curve:

I was an in-person lecture person, and the way I remember things is by associations. Like, I can remember where I was sitting and, like, what the professor was wearing when I learned which like, lecture material. So, like, sitting in the same spot, like, looking at the same computer screen, as I just scroll through notes, it’s really hard for me to associate that type of thing. So that was, like, the biggest adjustment. But now that we’ve been doing this so long, I think I’ve gotten better and just, like, found other resources and other ways to, like, find those associations. But the learning curve was real steep on that when we first went online. (S18)

Fourth, group dynamic was changed due to unfamiliarity with their peers. Compared with virtual CBL/TBL, in-person learning provides more opportunities to become familiar with each other academically (e.g., “knew our personalities how we worked and stuff” (S7)) and personally (e.g., “have more friendly chats” (S21)). They talked about significant differences between their 1st group (prior to COVID-19) and 2nd group (after COVID-19), for example, “We don’t collaborate anywhere near to the level we did with my original group” (S3). As such, group members’ individual personalities magnified how they participated in group interactions in the online setting. While quiet students tended to fall into the background more often, these who had strong opinions became more dominate during discussions, as explained in the following:

I definitely agree with you that the people who love to talk and share, they would do so more, and then those quiet people would tend to probably just go ahead and answer all the questions quietly instead of speaking up because sometimes probably it’s just hard for them to speak up when, when, you know that the other one is talking. And so, it’s easier for the quieter people to, to hold back. (S8)

Regarding the 2nd theme of learning communities, four codes emerged from the participants’ discussions: (1) interactions within the group, (2) interactions across different groups, (3) the roles of technology and TBL facilitators, and (4) impacts of the larger social context. In general, the participants felt overall virtual learning within the group deteriorated due to limited interactions and connections. While a few students highlighted greater self-study time and valued the efficiency of online CBL/TBL, a large majority of the participants believed that they lost the community. They reported that they “miss the in-person sort of camaraderie that we used to get and the atmosphere” (S26), and “don’t get to know each other more like, on a deeper level because there’s a total lack of connection between me and my, my new group” (S3). Team spirits disappeared in the online setting as described in the following:

I feel like the online, it’s now just 104 people in a room online, getting ready to answer these questions, whereas in person TBL, we had our teams. There was more of this sense of, you know, team spirit, we were all the different teams coming together to have the discussion. (S27)

Second, they believed learning across different groups was completely lost, as one described that “it’s harder to communicate with different groups, like, whereas in the room, maybe if you had a group at the table next to you, you could ask xxx question if your group didn’t understand it” (S11). The students also explained that they did not have a chance to see wrong answers or understand ambiguity between item distractors. Their social interactions were also very different prior to the pandemic “because everyone’s in the room like, you get to see people, like, you can get your coffee from the Starbucks in the school and, like, just kind of chat” (S22). As one student stated, “I guess the community aspect is lacking in that human interaction. So, on the social aspect, there isn’t that community” (S9). The following student described,

I feel like with us as a class, like, I truly miss seeing people in passing or seeing the people who went to lecture and talking to those people, instead of only the people that are in my PBL. I think there’s a lot of connections that have been lost since we’ve been online. Like, I don’t see the same, you know. There are people I would only see at school, and now I only see 5 people out of our class. So, I really miss, I miss our class. Like, I miss seeing everyone, so I feel like in that way learning has changed because I feel like my connections have kind of gone down. (S4)

Third, technology and how facilitators used technology competently impacted online TBL more significantly. The participants discussed “a weird period of navigating the new technology” (S3) and how multiple online systems (e.g., Teams, WebEx, and Top Hat) influenced CBL/TBL. In addition, as the students observed, online TBL became “more facilitator dependent” (S21). They said it felt “exhausting to go from a really good, well-prepared professor to someone who doesn’t know how to use [the technology]” (S13). Facilitators, as a result, did not always mange time effectively. Students experienced “people being pushed into the wrong groups, not getting into any group at all” (S18). Thus, “we’re not thoroughly discussing it, and we’re just like getting through it just to get through it and get an answer out” (S15).

Fourth, the educational, social, and political impacts of the larger context on online learning were enormous. The participants expressed their concerns about computer fatigue, COVID fatigue, and personal wellbeing. They were “worn out with the format and the computers” (S27), “so sick of being in the [living] room” (S2), struck in their “living space” (S9), and felt “life exhaustion” (S18). As the following student elaborated:

Like, it’s so hard to just sit in this chair, like, so long a day and study. So, like, I really liked in-person, just because it got me out of the house and got me somewhere different. I got to see other people and then I would be able to come home and just sit for, like, 4 hours, 5 hours and study here. But now class is here. [I’m] studying here in between classes. I go grab food and I bring food here. So, I sit in the same room probably like, 10 hours a day at least. (S3)

## Discussion

This study compared students’ perceptions about collaboration between face-to-face and online CBL/TBL. While two items in the quantitative survey showed significant differences, the focus group results provided much more in-depth information going beyond collaboration. Online learning offered students with greater autonomy and personal control over their learning; however, knowledge co-construction and group connections were impaired. On the one hand, this group of participants showed similar or even stronger personal accountability toward their learning processes and outcomes in the virtual setting. They took ownership of their learning and modified their learning strategies, which is consistent with the features of medical students who have been through selected admission processes and are generally considered self-motived learners [19]. On the other hand, the results showed significant decreases in collaboration due to the limitation of virtual communication, the shift towards efficiency, and the lack of knowledge sharing with their peers. The use of CBL/TBL is intended to develop the competency that groups of students work together to search for understanding, meaning, or solutions to medical problems or questions; however, opportunities to achieve this competency were minimized due to the limitations of the online setting within the current context.

More importantly, the study concluded that CBL/TBL involves more than collaborating to share knowledge and answer questions. The concept of a learning community emphasizes the importance of personal connection, mutual support, and a sense of belonging. Small groups provide an opportunity to foster learning communities that require a shared emotional connection to maximize learning expereinces [20, 21]. In a learning community, students interact and collaborate with each other, forming connections that extend beyond the classroom or course boundaries to develop social and cognitive skills and support each other emotionally. The results from this study showed that community building within and across groups were lost. Many participants perceived the missing learning communities in the virtual setting as well as technological challenges and the issue of technological competency of TBL facilitators. Participants elaborated on their isolations and expressed their concerns about the influence of the social, cultural, and political context. This is consistent with the social cultural learning theories that learning cannot be separated from the larger context [22, 23]. The learning communities that CBL/TBL could have been fostered in medical education were missing. Students not only lost personal connections with their group members in CBL/TBL, but also academic learning opportunities from other groups and connections with the community as a whole. The challenges that these medical students experienced in forming meaningful collaborations in the online settings are similar to those reported by students in other disciplines that have also made this transition [24]. In addition, faculty have a pivotal role in transitioning to virtual learning environments. Their adaptability in navigating different technology, proficiency with diverse software, and ability to cater to student needs during the virtual setting are all essential to create a successful and productive online learning experience. Their expertise not only in utilizing tools but also in fostering interactive and inclusive learning environments is integral to maximizing the potential of virtual learning.

Overall, virtual learning environments may have presented challenges for collaborative learning and the establishment of a sense of community. In face-to-face settings, students often have opportunities for informal interactions, networking, and building relationships within and beyond their immediate group. These interactions contribute to a richer learning experience and a sense of belonging within a larger learning community. However, in the virtual setting, such opportunities may have been limited, leading to a decreased sense of connection with other groups and the broader community.

## Conclusions and Limitations

Overall, the results of the study revealed noteworthy differences in terms of collaboration between face-to-face and virtual settings. Students in the virtual setting experienced a reduction in collaboration opportunities not only within their own group but also in terms of broader learning opportunities and social connections with other groups and the wider community. This study provides insights to inform future teaching and learning in undergraduate medical education. CBL/TBL can be used to create powerful learning communities that maximize learning, build cohesiveness, and counteract isolation. Medical schools and educators need to be cautious about adapting educational modalities to the virtual environment, as some of the benefits of collaboration and learning communities could fail to materialize. When adopting new technologies, educators should try to become as proficient with new software as possible to avoid disruptions to the learning process. In the ever-evolving landscape of the COVID-19 pandemic (or any potential future pandemics), educators will need to remain flexible and be mindful of unintended consequences of the various learning modalities. It is worth noting that the school under study opted to revert to in-person small group learning for the following year, partly influenced by the insights discussed in the paper. This decision reflects the complexities involved in balancing various instructional modalities in response to evolving circumstances.

There are some limitations to our study. First of all, the change from in-person to online might impact the way how students assessed their peers’ collaboration skills. Content differences in two courses may also impact how students collaborated with each other. This was a small-scale study focusing on only one educational context with its own characteristics regarding student population, learning approaches, and teaching features. It would be beneficial to extend the study by incorporating more data longitudinally from various courses, such as organ systems/foundations of medicine, and comparing the results between year 1 and year 2 preclinical courses. However, the school made decisions to switch back to in-person small group learning modalities in the following year. Given this change, it might be challenging to expand the study as originally planned. Perceptions may vary when using different populations in different educational contexts. Moreover, the participants in this study were likely to be mostly “virtual-naïve” as most students had limited exposure to online learning environments in their undergraduate education. The results might differ, if this study were conducted using newer students who have experienced the transition to online learning from COVID-19 as undergraduates. Also, one should not ignore the socio-cultural context of the earliest phase of the COVID-19 pandemic, when isolation and social distancing were new and widely used approaches that likely exacerbated the breakdown of collaborative relationships. Further research will be needed as the COVID-19 pandemic continues to evolve to determine how changing between virtual, face-to-face, and blended models of education affects students’ ability to collaborate and develop collaboration skills.

## Data Availability

This study is deemed exempt and anonymous data is available upon request from the authors.
